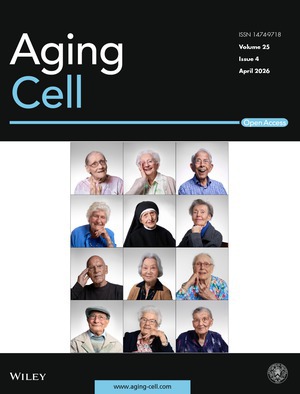# Featured Cover

**DOI:** 10.1111/acel.70473

**Published:** 2026-04-03

**Authors:** Flavien Delhaes, Justine Falciola, Adar Hoffman, Stéphanie Carnesecchi, Stefano Cavalli, Armin von Gunten, Daniela S. Jopp, François R. Herrmann, Karl‐Heinz Krause

## Abstract

Cover legend: The cover image is based on the article *Plasma Proteome Profiling of Centenarian Across Switzerland Reveals Key Youth‐Associated Proteins* by Flavien Delhaes et al., https://doi.org/10.1111/acel.70409.